# Hepatitis B Surface Antigen Could Contribute to the Immunopathogenesis of Hepatitis B Virus Infection

**DOI:** 10.1155/2013/935295

**Published:** 2013-01-16

**Authors:** Yasuteru Kondo, Masashi Ninomiya, Eiji Kakazu, Osamu Kimura, Tooru Shimosegawa

**Affiliations:** Division of Gastroenterology, Tohoku University Hospital, 1-1 Seiryo-Machi, Aoba-ku, Miyagi, Sendai City 980-8574, Japan

## Abstract

Various findings concerning the clinical significance of quantitative changes in hepatitis B surface antigen (HBsAg) during the acute and chronic phase of hepatitis B virus (HBV) infection have been reported. In addition to being a biomarker of HBV-replication activity, it has been reported that HBsAg could contribute to the immunopathogenesis of HBV persistent infection. Moreover, HBsAg could become an attractive target for immune therapy, since the cellular and humeral immune response against HBsAg might be able to control the HBV replication and life cycle. However, several reports have described the immune suppressive function of HBsAg. HBsAg might suppress monocytes, dendritic cells (DCs), natural killer (NK), and natural killer T (NK-T) cells by direct interaction. On the other hand, cytotoxic T lymphocytes (CTLs) and helper T (Th) cells were exhausted by high amounts of HBsAg. In this paper, we focused on the immunological aspects of HBsAg, since better understanding of the interaction between HBsAg and immune cells could contribute to the development of an immune therapy as well as a biomarker of the state of HBV persistent infection.

## 1. Introduction

Hepatitis B virus (HBV) is basically a noncytopathic DNA virus that causes chronic hepatitis and hepatocellular carcinoma (HCC) as well as acute hepatitis and fulminant hepatitis [1]. HBV now affects more than 400 million people worldwide and, in approximately 5% of adults and 95% of neonates who become infected with HBV, persistent infection develops [2]. HBV contains a small (3.2 kb), circular, partially double-strand DNA organized into four open-reading frames. The longest open-reading frame encodes the viral polymerase. The envelope open-reading frame is located within the polymerase open-reading frame in a frame-shift manner. The core and X open-reading frames partially overlap with the envelope open-reading frame [3, 4]. The covalently closed circular DNA (ccc DNA) is the template that is transcribed to generate four major RNA species: the 3.5 kb, 2.4 kb, 2.1 kb, and 0.7 kb viral RNA transcripts [5]. HBV produces Hepatitis B core antigen (HBcAg), Hepatitis B envelope antigen (HBeAg), Hepatitis B X antigen (HBxAg), and Hepatitis B surface antigen (HBsAg) that could contribute to the HBV life cycle. HBsAg was found by Blumberg et al. in 1965 and regarded as an HBV-related antigen in 1968 [6, 7]. The clinical significance of quantitative changes in HBsAg during the acute and chronic phase of HBV infection has been reported [8–10]. The amount of HBsAg has been found to be closely related to the activity of HBV replication in hepatocytes [8]. In addition to serving as a biomarker of HBV-replication activity, it has been reported that HBsAg could contribute to the immunopathogenesis of HBV persistent infection (Table 1) [11–17].

It has been shown that cellular immune responses including those of cytotoxic T lymphocytes (CTLs), Type 1 helper CD4+ T lymphocytes (Th1), FoxP3+ regulatory T lymphocytes (Tregs), and dendritic cells (DCs) play a central role in the control of virus infection [18–20, 28–34]. Moreover, Type 2 helper CD4+ T lymphocytes (Th2), B cells, and plasma cells could contribute to the production of HBV neutralizing and/or nonneutralizing antibody. Not only adaptive immune responses but also innate immune responses, including the intrahepatocyte innate immune response, and those of natural killer cells (NK), natural killer T cells (NK-T) and monocytes, might be involved in the coordination of all immune responses [15, 35–37]. Several reports have described that HBsAg could be involved in the orchestration of the immune responses, especially in disturbances of the appropriate immune responses [11, 14, 15, 38].

Recently, nucleos(t)ide analogs, such as lamivudine, adefovir, entecavir and tenofovir, and interferon-based therapy have been employed to control HBV [39, 40]. Unfortunately, the efficacy of nucleos(t)ide analogs is limited by viral reactivation through the emergence of escaped mutants in cases of prolonged treatment [39, 40]. Therefore, immunotherapy including interferon-based therapy is one of the significant options to eradicate or control HBV replication [41]. The aim of immunotherapy is to control the activity of HBV replication and to eradicate infected hepatocytes [42]. For this reason, the quantification of HBsAg and stimulation of HBsAg-specific immune responses might be important. It is necessary for the development of new strategies to understand the immuno-pathogenesis of HBV infection [43]. In this review, we focus on the immunological aspect of HBsAg based on various reports regarding HBsAg, lymphoid cells, and HBsAg-related immunotherapy.

## 2. HBsAg, T (CTLs, Th, Tregs, etc.) and B Lymphocytes

**Figure 1:**
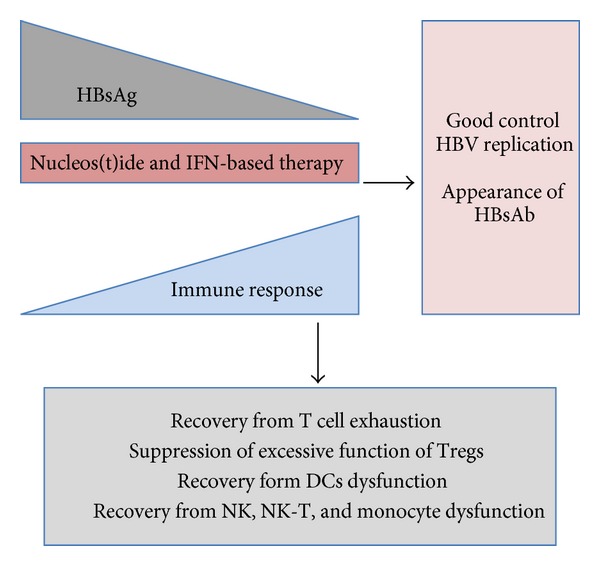
Scheme of recovery from immune suppression. The reduction of HBsAg could result in recovery from various kinds of immune suppression and possibly achieve resolution of HBV persistent infection or good control of HBV replication.

In the resolution of HBV infection, efficient recognition of the intracellular HBV antigens by the host immune cells is essential [32, 33, 44, 45]. It has been shown that the cellular immune system, including CTL, Th1, and Tregs, plays a central role in the control of virus infection. The hyporesponsiveness of HBV-specific CTL, Th1 cells, and excessive regulatory function of Tregs in peripheral blood have been reported in chronic hepatitis B (CHB) patients [2, 23, 24, 28, 29, 33, 46, 47]. Many groups including ours indicated that the treatment with nucleos(t)ide analogs in CHB could restore both CD4+ T cells and CTL hyporesponsiveness following the decline of serum levels of HBV-DNA and HBV-derived antigens (Figure 1) [20, 24, 46]. 

Although there are few reports indicating a direct suppression of the T cell immune response by HBsAg, we cannot exclude the possibility of a direct suppression of the T cell immune responses. It has been reported that T cell hyporesponsiveness in CHB might be induced by peripheral tolerance such as exhaustion [18, 20, 21, 30]. Recently, much attention has been devoted to the relevance of inhibitory receptor expression on T cells during chronic infections, including programmed death-1 receptor (PD-1) [22]. An HBV transgenic mouse model revealed the effect of PD-1 mediated T cell exhaustion [18]. 

CTLs recognize viral antigens synthesized within infected cells in the form of oligopeptides that are presented on HLA class I molecules [48]. Although the responses of HBV-specific CTLs are observed in a strong, polyclonal, and multi-specific way in patients with self-limited infection [47], hyporesponsiveness of HBV specific CTLs in the peripheral blood has been demonstrated in patients with HBV persistent infection. Various kinds of epitopes in HBV-core, surface, and polymerase were reported by many groups [48]. We also identified new, HLA-A24 restricted CTL epitopes in core and surface antigens [46]. Therefore, the amount of HBsAg in peripheral blood might influence the HBV-specific CTL response. 

Previously, we examined the mechanisms of hyporesponsiveness of HBV-specific CD4+ T cells by evaluating the Th1/Th2 commitment and activity of Tregs [23]. In CHB patients, HBsAg stimulation induced upregulation of GATA-3 mRNA compared with that in healthy volunteers, while the expression level of Th1-related mRNA remained unchanged. However, the suppression of either direction, to Th1 or Th2, by HBcAg stimulation was observed. HBcAg-specific Tregs produced IL10 and suppressed the immune response, while HBsAg stimulation favored Th2 deviation in CHB [23]. Negative regulation of CD8+ T cell responses during chronic HBV infection can also be mediated by immuno-suppressive cytokines such as IL10 and TGF-beta [29]. Recently, there has been increasing evidence indicating a role of Tregs in maintaining HBV infection [28, 29]. However, the relation between HBsAg and Tregs has not been clarified yet.

The extent to which the humoral immune response contributes to the control of chronic HBV infection is less clear. HBV-specific antibodies are indicators of certain stages of these diseases. HBsAg specific antibodies (HBsAb), detectable in patients who have recovered from acute HBV infection and in HBV-vaccinated individuals, serve as neutralizing antibodies that can inhibit viral attachment and entry [49]. The induction of HBsAb is sufficient to prevent infection. In CHB patients, seroconversion to HBsAb is considered to be a marker of disease resolution [50, 51]. Although B cells and plasma cells are important for producing HBsAb, there are few reports indicating direct suppression of the B cell functions by HBsAg in CHB patients. We need to consider the possibility of B cell dysfunction in CHB since the appearance of HBsAb during CHB treatment represents a favorable condition of the immune response, as mentioned above.

## 3. HBsAg, NK, and NK-T Cells

NK cells play a role in controlling the innate immune response during viral pathogen invasion in the early stage of infection, while regulating the adaptive immune responses in a persistently infected host. They are especially enriched in the liver where they comprise about 50% of the intrahepatic lymphocyte portion, as compared to the peripheral blood where they represent about 10% of the total lymphocyte population [52, 53]. NK cells can be detected by flow cytometry by the expression of CD56 and lack of CD3. Additionally, they can be separated into CD56^dim^ and CD56^bright^ cells. CD56^dim^ cells are considered to be mature NK cells that constitute majority of the population, whereas CD56^bright^ cells are the minority and thought to be at an early stage of maturation [35, 36]. They mediate the recognition and lysis of viral-infected cells and the production of immunoregulatory cytokines. Through the release of cytokines such as IFN-gamma GM-CSF, TNF-alpha TGF-beta IL-2, and IL-10, they may influence the adaptive immune system [54–56]. 

NK-T cells are included in a lymphocyte population identified with the expression of surface markers of NK cells together with T cell antigen receptor [57]. NK-T cells as well as NK cells are found markedly in the liver, with fewer found in the spleen or bone marrow [37]. NK-T cells produce large numbers of cytokines more quickly than NK cells. Therefore, NK-T cells have been considered to largely influence the innate and the adaptive immune response [58].

Little is known about the specific immune suppression of NK and NK-T cells by HBV-encoded antigens such as HBsAg. In 2005, Chen et al., showed that the number of hepatic NK cells was decreased with the expression of HBsAg and their cytotoxicity was attenuated in transgenic mice. In addition, the response of hepatic NK cells to specific stimulation by Poly (I:C) was changed and the increase in the antitumor cytotoxic activity of intrahepatic activated NK cells was markedly impaired [14]. However, in responders to vaccination with HBV, Alabarran et al. reported that NK and NK-T cells are effectively activated against HBsAg, as reflected in the elevated ratio of CD56^bright^ cells, increased NK-T cells, specific high levels of IL-2 and IFN-gamma intracellular cytokines in NK-T, and increase of IFN-gamma in NK cells. However, in nonresponders to vaccination, NK and NK-T cells showed an inactivation of capability and a diminished regulatory cytokine production [59].

## 4. HBsAg and Monocytes

Monocytes play roles in immune function and migrate from the bloodstream to other tissues to differentiate into tissue-resident macrophages or dendritic cells. They are characterized by the high expression of the CD14. Some groups have reported about the association between monocytes and HBV infection. Vanlandschoot et al., described that HBsAg suppressed the activity of monocytes and confirmed that recombinant HBsAg particles could bind almost exclusively to monocytes and that the binding to monocytes was enhanced by a heat-labile serum protein that was inhibited by Ca2M/Mg2M, low pH, and an HBsAg-specific monoclonal antibody. Moreover, the LPS and IL-2-induced production of cytokines was suppressed [15]. A study of the association between the human immune function and HBsAg using THP-1 cells, a human monocyte cell-line, indicated that HBsAg inhibits LPS-induced COX-2 expression and suggested that hepatitis B virus may regulate IFN-gamma production by inhibiting IL-18 and IL-12 production [13].

## 5. HBs-Ag and Dendritic Cells

**Figure 2:**
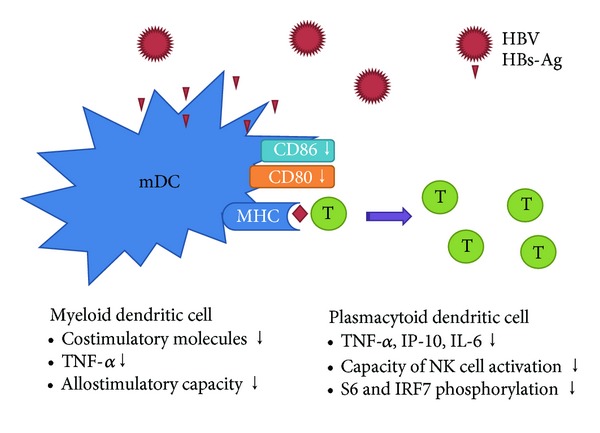
A schematic diagram of DC dysfunction in patients with HBV.

Dendritic cells are professional APC that initiate and mediate immune responses against pathogens and tumors. Typically, immature DCs capture and process antigens to peptides which are then presented in the context of MHC class II or class I molecules. They migrate to lymphoid tissues and present antigenic peptides to naive T cells. Previous studies demonstrated that the myeloid dendritic cells (mDCs) of patients with chronic HBV are indeed impaired in their capacity to mature compared to mDC of healthy controls, as shown by their decreased capacity to upregulate costimulatory molecules, produce proinflammatory cytokines, and stimulate T cells [12]. It has been also reported that monocyte-derived DCs and plasmacytoid DCs(pDCs) are functionally impaired by the presence of HBV [12, 25]. Concerning the inhibitory mechanism, whether HBV directly interferes with the DC function is not known, as only HBV DNA could be detected but no evidence was found for viral replication in circulating mDCs and pDCs [12, 26], nor in monocyte-derived DCs [27]. Thus, although HBV may be incapable of replicating in DCs, the binding and uptake of viral particles by these cells may be responsible for the impaired function of DCs in HBV-infected patients. Op den Brouw et al. reported that HBsAg is internalized by mDCs and inhibits the upregulation of costimulatory molecules on mDCs [11], and Woltman et al. reported that analyzing different HBV proteins revealed that HBeAg and especially HBsAg are involved in the suppression of pDCs function, and HBsAg abrogated the CpG-induced mTOR-mediated phosphorylation of S6, the subsequent phosphorylation of IRF7 and the transcription of IFN-*α* genes [60]. However, the direct immune regulatory effect of HBV and circulating HBsAg particles on the function of DCs can be considered as part of the mechanism by which HBV escapes immunity (Figure 2). 

## 6. HBsAg Contributing to Carcinogenesis and Immune-Suppression of HBV-Related HCC

Up to now, carcinogenesis of the HCC by HBV has been studied. HBV-associated carcinogenesis can be seen as a multifactorial process that includes a direct mechanism involving viral protein, indirect mechanisms through the chronic inflammation, and the integration of HBV DNA [61]. As for the direct mechanism of the viral protein, it has been reported that the HBx gene [62–65] and PreS2 gene [66] act as promoters of carcinogenesis, based on a transgenic mouse model and force expression model of cell lines. The Pre S2 protein is encoded by HBsAg. It activates mitogen-activated protein kinase (MAPK), which is a signal molecule that is involved in cell proliferation [67]. Moreover, PreS2 protein accumulates in the endoplasmic reticulum (ER) of hepatocytes, and DNA injury is caused in the cell by ER stress [68, 69]. These mechanisms are considered to be a cause of carcinogenesis. 

However, it is also thought that evasion from self-immunity is necessary for the growth of cancer. In recent studies, it was revealed that HBsAg carriers have 25–37 times increased risk of developing HCC as compared to noninfected people [70, 71]. Accordingly, it is thought that HBsAg functions in immune evasion, not only in promoting carcinogenesis. Actually, the accumulation of ER stress in hepatocytes causes the degeneration of protein, and evasion from self-immunity [72]. Moreover, it was reported that Pre S2 mutants increased hepatocellular carcinoma. These mutants reveal shorter forms of large, HBV surface antigens (LHBs), proteins with internal deletion. The deletion site (nucleotides 4–57) of Pre S2 has been recognized to correlate with an epitope of the CD8 T-cell response and B cell neutralization [33]. Therefore, Pre S2 mutants are involved in immune evasion. The immune evasion mechanism of HBV oncogenesis has not been fully elucidated, but further clarification is expected in the future.

It has been reported that the immune response could be suppressed by various kinds of mechanisms in HCC [73–81]. One of the important roles of immune suppression is induced by Tregs, as seen in HBV persistent infection [75–79, 82]. The frequency of Tregs infiltrating HCC was significantly higher than in non-HCC regions of the liver [83–86]. Moreover, HBV itself could induce excessive Tregs function [23, 24]. Therefore, the interaction between HBV and immune suppressive factors of HCC might strongly suppress cellular immune responses, including DCs, CTL, and Th1, and so forth, that are important for controlling HCC proliferation and HBV replication [87]. However, the relation between HBsAg expression and immune suppression in HCC is still unclear. In our ongoing study, the expression patterns of chemokines produced from several HCC cell lines with HBV replication were clearly different from those without HBV replication. Therefore, it is urgent to analyze the mechanisms of immune-suppression observed in HBV-related HCC.

## 7. HBsAg and Immunotherapy

In addition to HBcAg, HBsAg is one of the most important HBV-antigens that could induce HBV-specific cellular immune responses. HBsAg stimulation might easily induce Th2 immune responses. However, one group reported that HBsAg and CpG motif-containing oligodeoxynucleotides could induce Th1 immune responses [17]. Various kinds of HBsAg delivery systems were examined since the induction of a favorable immune response that could control HBV used to be difficult [17, 88–94]. Many groups have described that various kinds of recombinant hepatitis B vaccines could have a specific but transient effect on viral replication in HBsAg-positive CHB [95]. However, vaccines consisting of recombinant HBsAg and anti-HBs immunoglobulins could induce HBs-specific T cells efficiently since the formation of Ag-Ab immune complexes could be easily captured and taken up by DCs [96]. Although HBsAg might suppress the functions of DCs [11], HBsAg-pulsed DCs might enhance HBV-specific immune response in CHB patients [97]. 

## 8. Conclusion

HBsAg is not only a useful biomarker but also a protein that might suppress various kinds of immune cells contributing to innate and adaptive immune systems. Moreover, HBsAg could become an attractive target of immune therapy, since the cellular and humeral immune response against HBsAg might be able to control HBV replication and life cycles. Better understating of the interaction between HBsAg and immune cells could contribute to the development of immune therapy and a biomarker of the clinical state for HBV persistent infection.

## Figures and Tables

**Table 1:** Functions and the effect of HBsAg among the various kinds of lymphoid cells.

Lymphoid cells	Function	The effect of HBsAg for immune response	Reference
Innate immune response			
NK/NK-T cells	Independent of epitope cytotoxic function	Suppression of cytotoxic activity of Intrahepatic NK cells	[14]
	Cytokine secreation (IFN-gamma etc.)		
Monocyte	cytokine, chemokine expression	Suppression of monocyte activation	
		Suppression of LPS and IL-2 induced cytokines production	[13, 15]
Intrahepatocyte reaction (TLR signaling)	Detection of pathogen-associated molecular pattern	Unclear	
Adaptive immune response			
CD8+ CTL	HBV-specific cytotoxic function	CTL exhaustion/peripheral tolerance	[18–22]
	(Perforin, IFN-gamma, etc.)		
CD4+ Th cells	HBV-specific IFN-gamma secretion	Th2 commitment	[23]
			
Tregs	Immune suppresion via IL10 and/or cells to cell contact	Enhancing Tregs activity via stress-related proteins	[21, 23, 24]
Dendritice cells	HBV antigen presentation and secretion of cytokines	Inhibit the upregulation of costimulatory molecules on mDCs	[12, 25–27]
		Suppression of pDC function	
